# Tuberculosis of the Breast

**Published:** 1903-07-18

**Authors:** 


### TUBERCULOSIS OF THE BREAST.

ALTHOUGH tuberculosis of the breast is not a very common condition, yet it is met with sufficiently often to claim the practitioner's consideration, the more so on account of the difficulty of distinguishing the early stages of the disease from cancer. Tubercle of the mammary gland occurs most frequently in young women, and presents itself in three varieties: namely, as cold abscess, as the circumscribed nodule, and as disseminated tubercles. The disease is occasionally primary in the mamma, but more often it occurs secondary to tubercle in other organs. The difficulty in diagnosis may be considerable. In both diseases the upper and outer quadrant of the breast is the commonest situation of the tumour. In both, at first, the disease is painless, and in neither is the contour of the breast appreciably changed. The skin is in both conditions at first perfectly free over the swelling, not being thickened, adherent, or even red. The outline of the tumours is in each case ill-defined, gradually merging into the rest of the gland tissue. Chronicity usually characterises both, as does also the fact that the disease develops insiduously. Retraction of the nipple may be found in either disease. After a time the distinction may be easily made and tuberculosis diagnosed by the mass softening in the centre and breaking through the skin leaving, a sinus discharging tuberculous pus. The glands in the axilla in case of tubercle usually soon soften, suppurate and discharge. In cancer the axillary glands do not suppurate, nor do sinuses form. In tuberculous disease, the breast as a whole appears larger than its fellow, while in cancer the mamma is shrunken, so that it is smaller than the opposite gland. When the diagnosis is doubtful, and in the early stages it always is doubtful, it is quite unjustifiable to wait for changes to reveal the nature of the new formation. An incision should be made into the tumour without delay, and if it does not appear to be a cancer it should be removed and submitted to an efficient pathological examination. Every aseptic precaution should of course be taken. The course here proposed will clear up the diagnosis and enable the practitioner in the event of the disease being tubercle to give a much more favourable prognosis. As regards after treatment, dietetic and hygienic measures are most important, everything being done in fact to support the patient's general health, as in tuberculosis of other organs. The local treatment is surgical, and consists in opening cold abscesses, scraping, and disinfecting sinuses, removing circumscribed nodules, and excising the axillary glands. If the disease is of the disseminated variety with multiple sinuses, the advisability of removing the entire breast will have to be considered.

